# Haptic Nudging Using a Wearable Device to Promote Upper Limb Activity during Stroke Rehabilitation: Exploring Diurnal Variation, Repetition, and Duration of Effect

**DOI:** 10.3390/bs13120995

**Published:** 2023-12-02

**Authors:** Nada Signal, Sharon Olsen, Usman Rashid, Ruth McLaren, Alain Vandal, Marcus King, Denise Taylor

**Affiliations:** 1 Health and Rehabilitation Research Institute, Auckland University of Technology, Private Bag 92006, Auckland 1142, New Zealand; nada.signal@aut.ac.nz (N.S.);; 2 Centre for Chiropractic Research, New Zealand College of Chiropractic, Auckland 1060, New Zealand; 3 Department of Statistics, University of Auckland, 38 Princes Street, Auckland 1010, New Zealand; alain.vandal@auckland.ac.nz; 4 Callaghan Innovation, 5 Sheffield Crescent, Burnside, Christchurch 8053, New Zealand

**Keywords:** haptic nudging, wearable, stroke, upper limb, behaviour change, health technology, rehabilitation technology

## Abstract

Haptic nudging via wearable devices promotes physical activity and may increase upper limb movement in stroke rehabilitation. This study investigated the optimal approach to haptic nudging by examining diurnal variation, duration of effect, and repeated nudging. The study analysed data from a multiple-period randomised crossover study. A 12 h inpatient rehabilitation day was divided into 72 intervals in which participants with stroke (n = 20) randomly received either a ‘nudge’ or ‘no nudge’. Upper limb movement was observed, classified, and analysed using longitudinal mixed models. The odds of affected upper limb movement following a nudge compared with no nudge were significantly higher during active periods such as breakfast, lunch, and morning and afternoon activities (odds ratios (ORs) 2.01–4.63, 95% CIs [1.27–2.67, 3.17–8.01]), but not dinner (OR 1.36, 95% CI [0.86, 2.16]). The effect of nudging was no longer statistically significant at 50–60 s post-nudge. Consecutive delays in nudging significantly decreased the odds of moving when a nudge was eventually delivered. Contrary to expectations, people with stroke appear more responsive to haptic nudging during active periods rather than periods of inactivity. By understanding the optimal timing and frequency of haptic nudging, the design of wearable devices can be optimised to maximise their therapeutic benefits.

## 1. Introduction

The field of wearable technology has seen rapid development in recent years, particularly with respect to its application in promoting physical activity. Wearables are devices that integrate built-in sensors into accessories or clothing [1], such as wristbands [2], shoes [3], and sleeves [4], to monitor users’ movement and position. Sensor data are analysed to provide feedback about physical activity either through the device or via a mobile app on a smartphone or tablet [5]. When carried by the user, smartphones themselves can also act as wearable devices utilising their inbuilt sensors [6].

For those who are proactive about their health, the use of wearables to increase physical activity has become increasingly popular [7], with low- to moderate-quality evidence supporting their efficacy in healthy adults [5]. However, their potential to promote physical activity among those with clinical and age-related conditions is an area that warrants further investigation. Wearables offer a promising avenue for monitoring and promoting physical activity both within and beyond healthcare settings [8].

As a leading cause of disability worldwide [9], stroke is a clinical condition that could benefit from wearable devices. Following stroke, rehabilitation incorporating high doses of task-specific physical activity is recommended [10,11], yet people with stroke often receive limited rehabilitation and have very low levels of physical activity [12,13]. While wearables have been used to encourage physical activity after stroke, and their potential to increase locomotor and upper limb activity is recognised by both physical therapists and people with stroke [14], the evidence for their efficacy is limited. A 2018 Cochrane review reported that wearables demonstrated no clear effect on locomotor activity after stroke [15]. However, one randomised controlled trial in people with stroke demonstrated that wearable step count monitoring increased physical activity when combined with additional behavioural change strategies such as an exercise calendar, goal setting, and praise for achievement [16].

The combination of physical activity monitoring via wearables with strategies that support behaviour change has been emphasised as an approach that may improve their efficacy [5,17,18]. Such strategies might include feedback about progress and goal attainment, rewards, coaching, social support, and nudging [18,19]. Nudging refers to the concept of manipulating choice architecture to influence decision making and behaviour [20]. Nudging has been shown to be effective at promoting physical activity in both healthy people and those with health conditions [21,22,23]. One nudging approach commonly used within wearable technologies to prompt physical activity is haptic stimulation. Haptic nudging is delivered using a small vibratory motor inside a wearable technology. Users are encouraged to respond to haptic nudging by performing a specific behaviour, for instance, completing rehabilitation exercises or resuming physical activity [24].

Within stroke rehabilitation, where clinicians seek to promote active movement in the affected upper limb [12,25], observational and feasibility studies have explored the use of haptic nudging to prompt the use of the affected upper limb [26,27]. These studies have shown that haptic nudging is not only feasible in people with stroke [26] but can also significantly increase the likelihood the user will move their affected upper limb following haptic nudging [27]. Prior to conducting larger clinical trials to establish the efficacy of wearable devices combined with behavioural change strategies to enhance upper limb recovery, the optimal method of delivering haptic nudging to promote physical activity in people with stroke should be explored. The following study utilised the BuzzNudge wearable device, which is a wrist-worn haptic nudge system that prompts users with stroke to move their affected upper limbs. Using data from a single-day inpatient stroke rehabilitation trial in which participants wore the BuzzNudge, this study explored the following research questions: (i) Is there diurnal variation in the effect of a haptic nudge? (ii) How long does the effect of a haptic nudge last? (iii) Is the effect of a haptic nudge dependent on the repetition of nudges?

## 2. Materials and Methods

### 2.1. Dataset

The dataset analysed in this study is from a multiple-period randomised crossover study in which 20 people undergoing inpatient stroke rehabilitation received haptic nudge prompts over the course of a single day via the BuzzNudge wearable device. The BuzzNudge is a Bluetooth-enabled wearable device with a 2.3 V coin vibration motor (Precision Microdrives Ltd., London, U.K., Model 310–103) that delivers three consecutive vibratory stimuli (0.3 s duration at 150 Hz) within 1.5 s. Haptic nudges were prompting in nature; participants were informed of the value of moving the affected upper limb after stroke and instructed to “move, try and move, or visualise moving their (affected) arm” following a nudge. The primary aim of the study was to evaluate the effect of haptic nudges on the observed amount of upper limb movement, and the primary results of the trial have been published elsewhere [27]. Ethical approval (16/NTA/74) was obtained from the New Zealand Health and Disability Ethics Committees, and the trial was registered (ACTRN12616000654459) with the Australia New Zealand Clinical Trials Registry. All participants provided written informed consent before data collection. Details of the data collection procedures pertinent to the current analysis are summarised here for the reader’s convenience.

### 2.2. Study Procedures

Twenty people with stroke who had an upper limb disability were recruited to the study. Relevant demographic, clinical, and medical information were gathered from the medical records of participants who gave their consent. The information obtained encompassed age, sex, ethnicity, stroke classification and date, the side of the body affected, dominant hand before the stroke, admission date, anticipated discharge date, underlying health conditions, and prescribed medications. 

**Figure 1:**
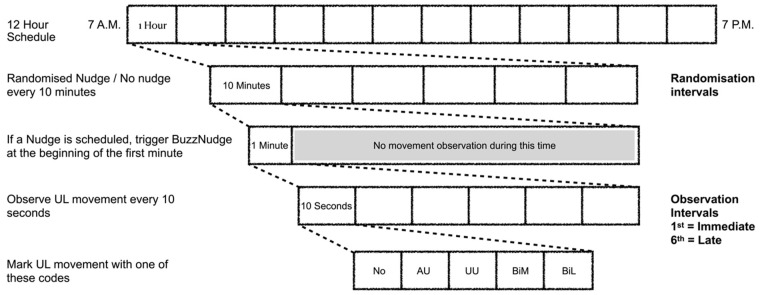
Data collection protocol. UL: upper limb. No: no upper limb (UL) movement. AU: affected UL movement. UU: unaffected UL movement. BiM: bimanual movement. BiL: bilateral movement.

Data were collected for a 12 h period on a single day of inpatient rehabilitation (7.00 a.m.–7.00 p.m.). The 12 h day was divided into 72 10 min intervals (referred to as randomisation intervals). For each participant, during 36 of these 72 randomisation intervals, a haptic nudge was delivered. The data collection protocol is shown in Figure 1. At the beginning of each 10 min interval (or as early in this period as was practicable), observational data were collected for 1 min. If a haptic nudge was scheduled, it was manually triggered at the beginning of this 1 min period. A trained clinical researcher recorded the participant’s upper limb activity during each 10 s interval of the 1 min period (referred to as *observation intervals*). Volitional movement was categorised in accordance with a previously published protocol and taxonomy [12,27,28]. In brief, UL movement was defined as follows:

- No movement, where no movement was observed;
- Affected upper limb movement, where movement of the affected upper limb alone was observed;
- Unaffected upper limb movement, where movement of the unaffected upper limb alone was observed;
- Bimanual movement, where movement of both upper limbs was observed to perform a common task;
- Bilateral movement, where movement of both upper limbs was observed to perform unrelated tasks [12,28].

Thus, over the 12 h period, 432 movement observations were recorded for each participant.

### 2.3. Haptic Nudge Randomisation Schedule

To evaluate and account for potential carry-over effects, six types of nudge sequences were used as the basis for the randomisation schedule. These sequences were as follows:

- Nudge;
- No nudge;
- Nudge-Nudge;
- No nudge-No nudge;
- Nudge-Nudge-Nudge;
- No nudge-No nudge-No nudge.

Where ‘Nudge-Nudge’ indicates that the participant was scheduled to receive haptic nudge prompts in two consecutive 10 min randomisation intervals. In contrast, ‘No nudge-No nudge-No nudge’ indicates that the participant was scheduled not to receive haptic nudge prompts for three consecutive randomisation intervals. For each participant, each nudge sequence appeared six times in their randomisation schedule. The randomisation schedule was also balanced so that during each of the randomisation intervals, 10 participants were scheduled to receive a nudge, and 10 participants were scheduled to receive no nudge.

### 2.4. Data Analysis

#### 2.4.1. Statistical Analysis

Deviations from the randomisation protocol occurred when a scheduled nudge was missed for various reasons (e.g., the participant was sleeping) or when a nudge was occasionally given in error. There was a loss of observation data due to drop-outs (n = 2 participants) and missing observations (354 out of 7776 observations) when the participant was not visible to the observer. Thus, the dataset had the potential to be unbalanced [29,30]. Consequently, traditional methods of analysis were deemed inappropriate [31], and longitudinal mixed models were used along with data imputations based on the worst outcomes in the dataset [32,33,34]. As the purpose of the current analysis was to understand the time and repetition dependence of the haptic nudge effects, it was important to account for the received nudges rather than the scheduled nudges and the deviations from the randomisation schedule. Therefore, to counter the effect of unobserved confounding variables, an instrumental variable (IV) analysis was adopted [35,36] to allow for the estimation of a complier average causal effect (CACE). A detailed description of the statistical modelling is presented in the Supplementary File using the R environment and package *lme*4*, ggplot*2*, ggpubr, emmeans, dplyr, forcats,* and *splines* [37,38,39,40,41,42,43,44]. 

#### 2.4.2. Estimated Effects

To determine whether there was diurnal variation in the effect of haptic nudging, five different time periods were selected for analysis based on the rehabilitation ward routine reflecting meal and therapy times: breakfast (8:00 a.m.–9:00 a.m.), morning therapy activity (10:30 a.m. to 11:30 a.m.), lunch (12:00 p.m. to 1:00 p.m.), afternoon therapy activity (1.30 p.m. to 2.30 p.m.), quiet period (3:30 p.m. to 4:30 p.m.), and dinner (5:00 p.m. to 6:00 p.m.). The effects of nudging during these time periods were estimated during the immediate (1st) 10 s observation interval after nudge delivery and were expressed as odds ratios, where the odds ratio reflects the ratio of the odds of affected UL movement during the observation period following a nudge to the odds of affected UL movement during the observation period following no nudge. A pairwise comparison of the odds ratio of activity periods and odds ratio for ‘Day’ was undertaken using constructing ratios of odds ratios. In addition, to demonstrate the variation in the immediate response to haptic nudges across the day, the probability of affected upper limb movement following a single nudge or no nudge was plotted over the 12 h day. 

To determine the duration of the effect of haptic nudging, the change in log odds of affected upper limb movement across observation intervals was calculated. To determine the effect of the repetition of haptic nudges, the change in log odds of affected upper limb movement in response to consecutive repetitions of a nudge or consecutive delays before a nudge was eventually delivered was calculated. These effects were estimated as log-linear trends. The estimated effects were reported along with standard errors, 95% confidence intervals, and hypothesis tests for null effects. Statistical significance was set at 0.05. An online tool was also made available that can be used to evaluate upper limb movement in relation to different combinations of repetitions of nudge, time of the day, and other variables in the dataset (https://gallvp.shinyapps.io/buzznudge_viz accessed on 1 December 2023). 

## 3. Results

Twenty people with stroke consented to participate in the research, as detailed in Table 1. The cohort’s median age was 76 years (IQR: 68–83 years), and the median duration post-stroke was 23.5 days (IQR: 8.25–38.25 days). Of these participants, 9 exhibited left hemiparesis, 10 right hemiparesis, and 1 bilateral symptom. Stroke syndromes were classified as follows: 5 cases of total anterior circulation syndrome, 10 of partial anterior circulation syndrome, 4 of lacunar syndrome, and 1 of posterior circulation syndrome. Attrition included two participants, one due to technical difficulties and the other owing to anxiety precipitated by the use of the wearable device.

### 3.1. Is There Diurnal Variation in the Effect of a Haptic Nudge? 

**Figure 2:**
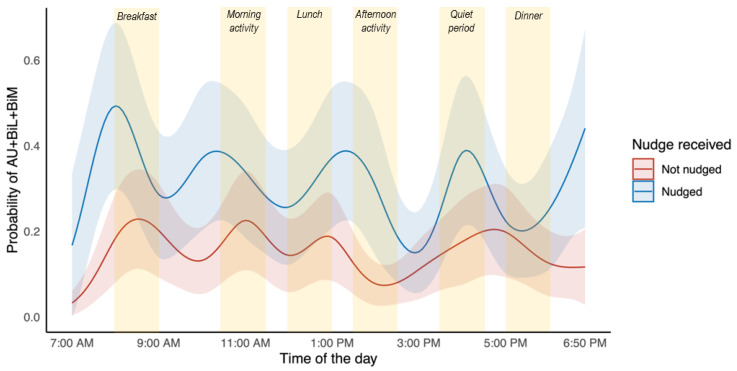
Probability of affected UL movement with 95% confidence intervals during the 1st observation interval as a function of time of the day and whether the participant received a nudge (blue line) or no nudge (red line). Selected time periods that were analysed are shaded yellow. AU: affected upper limb (UL) movement; BiL: bilateral movement; BiM: bimanual movement.

An illustration of the probability of affected upper limb movement across the day relative to whether the participant was nudged or not is presented in Figure 2.

The single nudge effects estimated from immediate observation intervals (first 10 s of first 1 min following nudge) at various times of the day and the daily average are provided in Table 2. These results suggest that for all selected time periods, except dinner, the odds of moving the affected upper limb movement were significantly higher immediately following a nudge than when the participant was not nudged. In addition, pairwise comparisons revealed these nudging effects were significantly greater during the afternoon activity compared to the daily average (*p* = 0.001) and were significantly lower during the dinner period compared to the daily average (*p* = 0.001). 

### 3.2. How Long Does the Effect of a Haptic Nudge Last?

**Figure 3:**
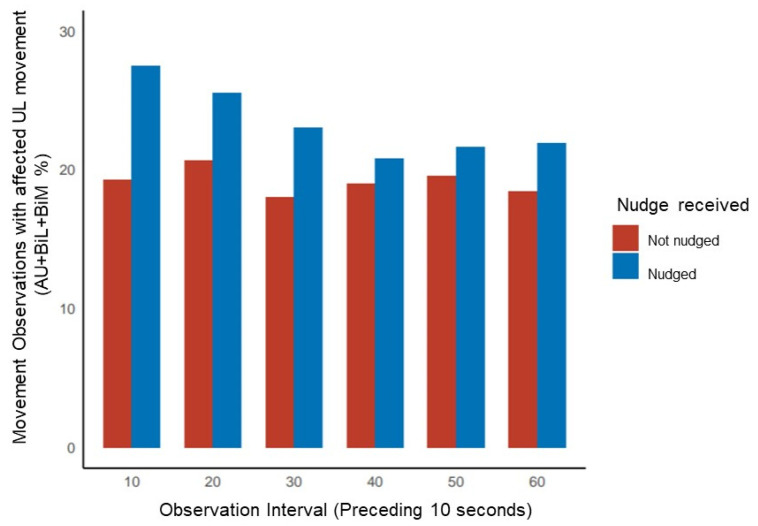
Proportion (%) of movement observations with affected UL movement across the 1 min observation period in 10 s intervals. AU: affected upper limb (UL) movement; BiL: bilateral movement; BiM: bimanual movement.

Across the day, the odds of affected upper limb movement following a nudge versus no nudge in the first 10 s observation interval was 2.37 (95% CI [1.68, 3.34], *p* < 0.001). In contrast, by the 50–60 s observation interval, the odds of affected upper limb movement following a nudge versus no nudge was 1.37 (95% CI [0.97, 1.93], *p* = 0.073). Across the 1 min observation period following a nudge, the odds of affected upper limb movement decreased significantly with each subsequent 10 s interval (−0.11 ± SE 0.03, 95% CI [−0.17, −0.06], *p* < 0.001). An illustration of the proportion of movement observations where the affected upper moved with respect to nudging is presented in Figure 3. 

### 3.3. Is the Effect of a Haptic Nudge Dependent on the Repetition of Nudges? 

The change in log odds of affected upper limb movement for consecutive repetitions of a nudge (e.g., Nudge-Nudge, Nudge-Nudge-Nudge) or consecutive delays before a nudge are listed in Table 3. These results suggested that with consecutive repetitions (e.g., Nudge-Nudge versus Nudge, or Nudge-Nudge-Nudge versus Nudge-Nudge), the odds of affected upper limb movement did not increase significantly. However, with consecutive delays in nudging (e.g., No nudge-No nudge-Nudge versus No nudge-Nudge), the odds of affected upper limb movement decreased significantly for each consecutive delay before a nudge (*p* = 0.0003).

## 4. Discussion

The aim of this study was to explore the optimal method of delivering haptic nudging to promote affected upper limb activity in people with stroke by investigating the temporal effects of haptic nudging. The study findings demonstrate that in people with stroke undertaking inpatient rehabilitation, the effectiveness of haptic nudging is influenced by various factors such as the time of day, the duration of effect, and the repetition of nudging. 

### 4.1. Effect of Haptic Nudging

#### 4.1.1. Diurnal Variation

The BuzzNudge wearable device was designed to address the inactivity of the affected upper limb, and it had been presumed that haptic nudging would be most effective during periods of inactivity rather than high activity periods. However, our findings contradict this assumption and indicate that people with stroke are more responsive to haptic nudging during periods of activity. The odds of moving the affected arm were significantly greater when haptic nudges were delivered during the afternoon activity period (OR 4.63, 95% CI [2.67, 8.01]) than when delivered over the entire day (OR 2.37, 95% CI [1.68, 3.34]); thus, nudging during the afternoon activity period almost doubled the effects of the haptic nudge. The afternoon period in this specific rehabilitation ward included either group exercise sessions or 1:1 therapy. The least effective time to deliver nudges was at dinner (5.00–6.00 p.m.), which may reflect fatigue at the end of the day [45]. Interestingly, nudges were still effective during the afternoon quiet period (3.30–4.30 p.m.), where the odds of affected upper limb movement were 2.5 times the odds when not nudged. It is worth noting that although this period was considered a quiet period for rehabilitation, the presence of family and friends may have encouraged the participants to respond to the nudges. 

In this study, participants received 36 nudges over the day. Other investigators have reported that people with stroke prefer hourly prompts [26,46]. However, in one pilot study [47], participants were required to respond to haptic nudging by performing activities from a task-based rehabilitation program [48], suggesting that the required behavioural burden in response to a nudge was far greater than in our study. If fewer nudges are preferred, careful consideration of the required behavioural response and the timing of the nudge with respect to other activities is needed. Our study suggests that haptic nudges coinciding with high activity periods may be more effective for individuals in an inpatient setting, while late afternoon and evening should be avoided. However, these patterns may vary depending on the healthcare setting and the individual’s usual daily routine. 

Observational studies of upper limb activity indicate that upper limb movement usually involves both limbs in the form of bimanual and bilateral activity [49,50]. Therefore, in the context of upper limb rehabilitation, the purpose of haptic nudging should be to incorporate the affected limb into an activity that can be performed bimanually or bilaterally. This differs from haptic nudging to promote lower limb activity, such as locomotion, where the activity can only be performed with both limbs. Understanding this distinction is critical for the design of wearable devices for upper limb stroke rehabilitation. To effectively prompt the integration of the affected upper limb into bimanual and bilateral tasks, devices applied to both upper limbs may be required. 

#### 4.1.2. Duration of Effect

Our study found that the duration of the haptic nudge effect was less than 1 min. This finding aligns with the nudge literature, which differentiates between nudges that prompt an immediate behaviour and those that bridge time [51] but contrasts with the findings of Da-Silva et al. [47], who reported larger volumes of activity in the subsequent hour. In our study, haptic nudging may have induced an immediate response by temporarily altering a person’s accessible thoughts (e.g., by making them aware of their stroke-affected arm) or by momentarily directing bounded attention [51]. For example, when already performing a task, nudging may have prompted the person to integrate their affected upper limb into the task. For nudging to induce lasting effects, it is likely that other strategies, such as social accountability, planning, and changing attitudes and beliefs, are required [51]. In our study, participants were instructed to “move your arm” when nudged. However, the motor learning [52] and behavioural change [53] literature would suggest that nudges might be more effective if explicitly tied to activities that are meaningful and enjoyable for the person. Therefore, future research should explore the provision of more personalised instructions and additional behavioural change strategies, which could be facilitated through a mobile interface.

#### 4.1.3. Repetition of Nudges

The findings indicate that nudges delivered in succession did not have a cumulative effect. However, longer breaks without haptic nudging resulted in a diminished effect when a nudge was eventually delivered. This suggests that an absence of stimulation can cause a decline in attention to upcoming stimuli. Therefore, when re-initiating nudging after a period of rest, a series of repeated nudges should be provided. In terms of the ongoing effect of repeated nudges, the habituation literature would suggest that over time, the response to frequent stimulation might decrease as the person becomes desensitised [54]. In this study, this desensitisation effect was not observed; however, the study was limited to one 12 h day. Further research is needed to investigate the response to repeated nudging over longer periods of time. 

### 4.2. Limitations

The data utilised in this study were obtained through the observation and the recording of movement by a researcher who was also responsible for triggering the nudge. Although the participants were unaware of the study hypothesis, the research protocol, including the observation by the researcher, could have influenced their response to the haptic nudge. A more comprehensive evaluation of individuals’ sensorimotor, perceptual, cognitive, and communication impairments, as well as their upper limb functional abilities, could have provided a more nuanced interpretation of the effect of haptic nudging in people with different clinical presentations of stroke. Furthermore, a primary limitation of this study was its reliance on secondary data analysis, which did not involve predetermined research questions and analysis methods, potentially introducing bias and limiting the effectiveness of data analysis.

### 4.3. Future Directions

The findings of this study provide valuable insights for clinicians, designers, and engineers involved in the development and evaluation of wearable devices and mobile applications that utilise haptic nudging to promote physical activity in people with stroke. Future research should focus on the development of wearable devices and haptic nudging approaches that can reliably monitor bilateral and bimanual upper limb movements to ensure that nudging effectively supports the incorporation of the affected upper limb into daily activities. Moreover, the design of such devices and approaches should account for factors like fatigue and impairments in sensory, perceptual, and cognitive systems to facilitate translation to clinical populations. Additionally, studies should investigate how haptic nudging can be combined with other behaviour change techniques to promote long-lasting effects and should be conducted over more extended periods to assess their long-term efficacy. Future research can build on these findings and address these important research gaps to improve the efficacy of haptic nudging approaches in promoting physical activity and functional recovery in people with stroke.

## 5. Conclusions

Haptic nudging can effectively promote movement of the affected upper limb after stroke; however, the findings of this study indicate that individuals appear to be more responsive to nudging during periods of high activity and less responsive at the end of the day. This challenged our assumptions about how the device might be integrated into clinical practice. The findings of this study demonstrated that the effect of a single haptic nudge was highest in the first 10 s following the stimulus and returned to baseline by 1 min. The effect of a single nudge was diminished when provided following a period of no nudging. Further research is needed to determine the effects of different haptic nudge schedules over longer periods of time.

## Figures and Tables

**Table 1:** Participant characteristics.

Participant	Age Range (Years)	Sex	Stroke Classification	Days Since Stroke	Affected UL	Affected UL = Dominant Hand
1	70–79	Male	LACS-I	9	Left	No
2	80–89	Female	TACS-I	39	Left	No
3	70–79	Female	TACS-I	59	Left	No
4	40–49	Female	LACS-H	8	Right	Yes
5	60–69	Female	PACS-I	5	Right	Yes
6	80–89	Male	PACS-I	34	Left	No
7	70–79	Male	TACS-I	27	Right	Yes
8	80–89	Female	PACS-I	7	Left	No
9	80–89	Male	TACS-I	67	Right	Yes
10	60–69	Male	PACS-I	36	Right	Yes
11	50–59	Female	TACS-I	25	Left	Yes
12	70–79	Male	LACS-H	33	Left	No
13	80–89	Male	PACS-I	12	Left	No
14	60–69	Male	LACS-I	3	Right	Yes
15	70–79	Male	PACS-H	40	Right	Yes
16	80–89	Female	PACS-I	22	Right	Yes
17	60–69	Female	PACS-I	6	Left	No
18	60–69	Male	POCS-I	10	Bilateral	Yes
19	80–89	Male	PACS-I	9	Right	Yes
20	80–89	Male	PACS-H	160	Right	Yes

UL: upper limb; LACS-I: lacunar circulation syndrome ischaemic; TACS-I: total anterior circulation syndrome ischaemic; LACS-H: lacunar circulation syndrome haemorrhagic; PACS-I: partial anterior circulation syndrome ischaemic; PACS-H: partial anterior circulation syndrome haemorrhagic; POCS-I: posterior circulation syndrome ischaemic.

**Table 2:** Single nudge effects from immediate observation intervals at selected times of the day.

Time of the Day	Odds Ratio ± SE [95% CI]	Z-Value	*p*-Value	Pairwise Comparisons against Odds Ratio for ‘Day’ *p*-Value
Day (7.00 a.m.–7.00 p.m.)	2.37 ± 0.41 [1.68, 3.34]	4.95	<0.001 *	
Breakfast (8.00–9.00 a.m.)	2.58 ± 0.66 [1.57, 4.26]	3.72	<0.001 *	0.614
Morning activity (10.30–11.30 a.m.)	2.01 ± 0.47 [1.27, 3.17]	2.99	0.003 *	0.354
Lunch (12.00–1.00 p.m.)	2.17 ± 0.53 [1.35, 3.49]	3.20	0.001 *	0.617
Afternoon activity (1.30–2.30 p.m.)	4.63 ± 1.29 [2.67, 8.01]	5.47	<0.001 *	0.001 *
Quiet period (3.30–4.30 p.m.)	2.51 ± 0.67 [1.49, 4.22]	3.45	<0.001 *	0.769
Dinner (5.00–6.00 p.m.)	1.36 ± 0.32 [0.86, 2.16]	1.30	0.194	0.001 *

Odds ratio is defined as the odds of affected upper limb movement when nudged divided by the odds of affected upper limb movement when not nudged; SE: standard error; CI: confidence interval. * *p*-value < 0.005, where the null hypothesis is that the odds ratio is equal to 1.

**Table 3:** Log-linear trends estimated for consecutive repetitions of a nudge or consecutive delays before a nudge.

Nudge Pattern	Log Odds/Consecutive Condition ± SE [95% CI]	Z-Value	*p*-Value
Repetition	−0.1 ± 0.1 [−0.3, 0.1]	−0.9	0.4
Delay	−0.4 ± 0.1 [−0.5, −0.2]	−3.6	0.0003 *

Log odds change represents whether the odds of affected upper limb movement changed in response to the repetition or delay of nudges; SE: standard error; CI: confidence interval. * *p*-value < 0.005, where the null hypothesis is that the log-linear trend is equal to 0.

## Data Availability

Ethical restrictions prevent public data sharing.

## Supplementary Materials

The following supporting information can be downloaded at: https://www.mdpi.com/article/10.3390/bs13120995/s1.
