# Supplementary material for: Haptic Nudging Using a Wearable Device to Promote Upper Limb Activity during Stroke Rehabilitation: Exploring Diurnal Variation, Repetition, and Duration of Effect
Source: Behav Sci (Basel). 2023 Dec 2;13(12):995. doi: 10.3390/bs13120995 (PMC10740938; doi:10.3390/bs13120995)
Supplement: Supplementary file 1 [file behavsci-13-00995-s001.zip › behavsci-2685653-supplementary.pdf]

Supplemental: Exploring diurnal variation, repetition, and duration of effect for haptic nudging

Usman Rashid

05/06/2023

Contents

1 Variables Included in the Analysis 2

2 Data Visualisations 3
2.1 UL Movement Distribution . . . 3
2.2 Participant-wise Proportions for AU+BiL+BiM . . . 4
2.3 Hour-wise AU+BiL+BiM Proportions across the Day . . . 5
2.4 AU+BiL+BiM Proportions for Movement Observations over 10-second Intervals . . . 6

3 Instrumental Variable Analysis 7
3.1 Stage I: Confounder Estimation . . . 7
3.1.1 Longitudnal Mixed Models . . . 7
3.2 Stage II: CASE Estimation . . . 8
3.2.1 Data Imputations . . . 8
3.2.2 Longitudnal Logistic Mixed Model . . . 8

4 Results 9
4.1 Immediate Effects of a Single Haptic Nudge on AUL Movement . . . 9
4.1.1 Immediate Effects over the Day . . . 11
4.2 Late Effects of a Single Haptic Nudge on AUL Movement . . . 12
4.2.1 Late Effects over the Day . . . 13
4.3 Effects of Repeated Nudges . . . 14
4.4 Effects of Deferred Nudges . . . 16

1 Variables Included in the Analysis

```
## 'data.frame':    8640 obs. of  14 variables:
##  $ ParticipantID                : Factor w/ 20 levels "1","2","3","4",..: 1 1 1 1 1 1 1 1 1 1 ...
##  $ Hour                         : Factor w/ 12 levels "1","2","3","4",..: 1 1 1 1 1 1 2 2 2 2 ...
##  $ Time.labels                  : Factor w/ 72 levels "1:00 PM","1:10 PM",..: 55 56 57 58 59 60 61
##  $ Ten.minute.interval          : num  1 2 3 4 5 6 7 8 9 10 ...
##  $ Ten.second.interval          : num  1 1 1 1 1 1 1 1 1 1 ...
##  $ Nudge.status                 : Factor w/ 4 levels "Nudged","Not nudged",..: 3 2 1 3 1 2 2 2 1 1
##  $ Nudge.scheduled              : Factor w/ 2 levels "Scheduled nudge",..: 1 2 1 1 1 2 2 2 1 1 ...
##  $ Nudge.received               : Factor w/ 2 levels "Nudged","Not nudged": 2 2 1 2 1 2 2 2 1 1 ...
##  $ More.of.the.same.scheduled    : num  0 0 0 1 2 0 1 2 0 1 ...
##  $ More.of.the.opposite.scheduled: num  0 0 0 0 0 2 0 0 2 0 ...
##  $ More.of.the.same             : num  1 1 0 0 0 0 1 2 0 1 ...
##  $ More.of.the.opposite         : num  0 0 1 0 0 0 0 0 2 0 ...
##  $ UL.movement                  : Factor w/ 6 levels "No","AU","UU",..: 6 1 3 6 1 1 3 3 3 3 ...
##  $ Combined.UL.movement         : Factor w/ 2 levels "UU+No","AU+BiL+BiM": NA 1 1 NA 1 1 1 1 1 1 ..
```

2 Data Visualisations

2.1 UL Movement Distribution

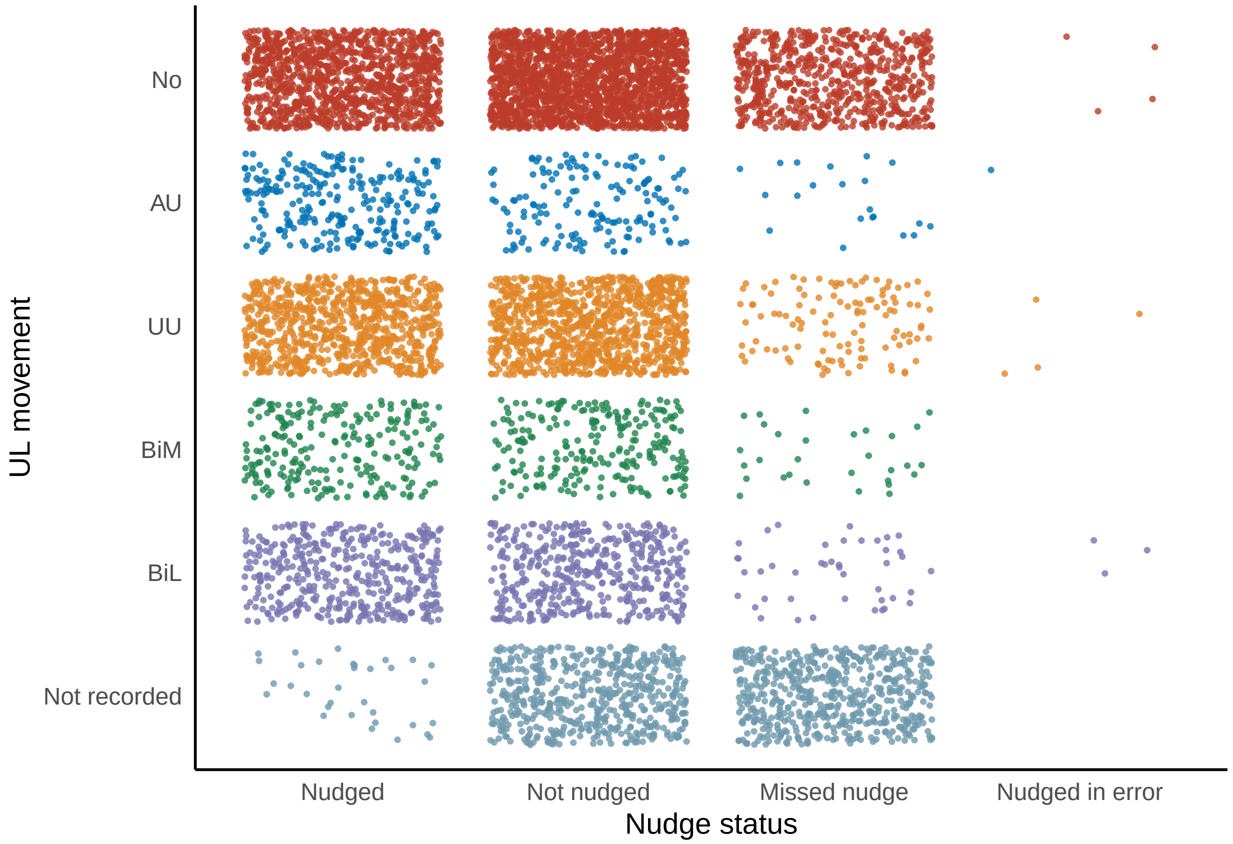

2.2 Participant-wise Proportions for AU+BiL+BiM

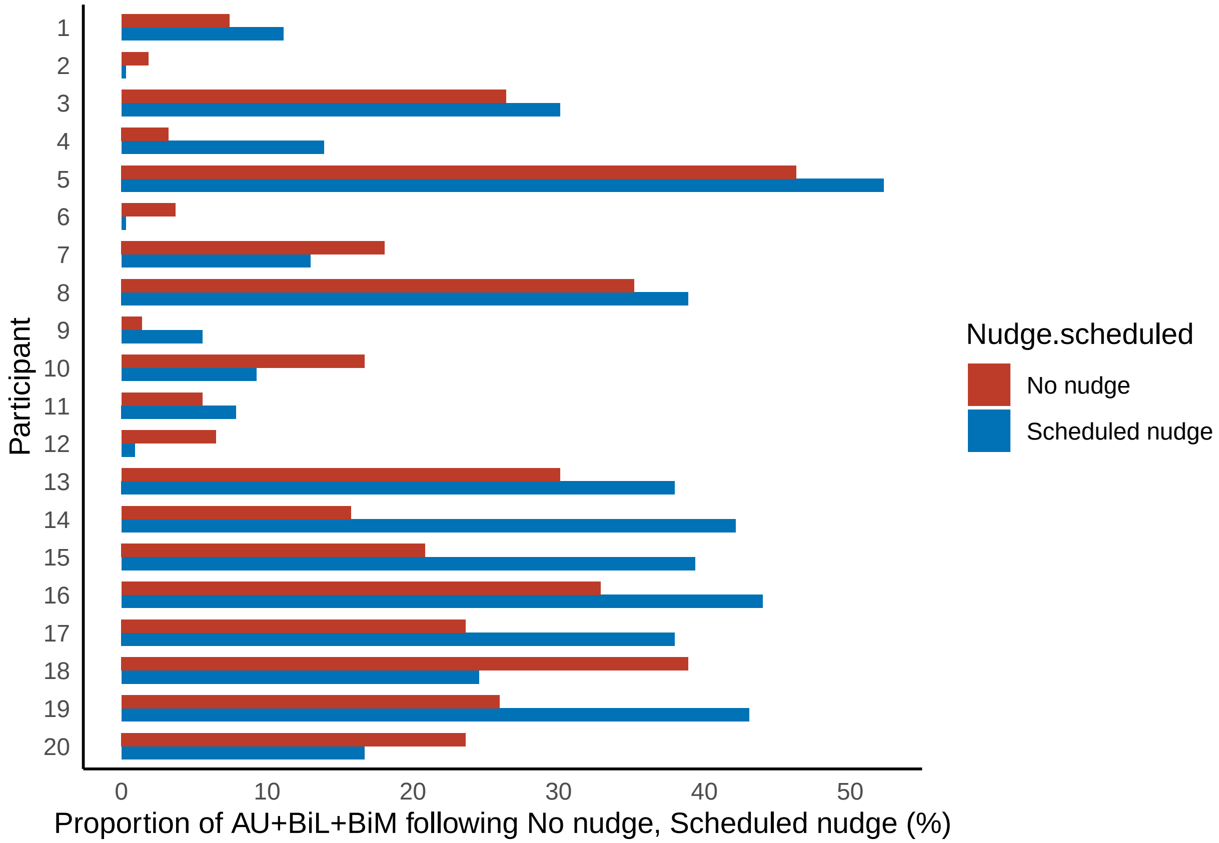

2.3 Hour-wise AU+BiL+BiM Proportions across the Day

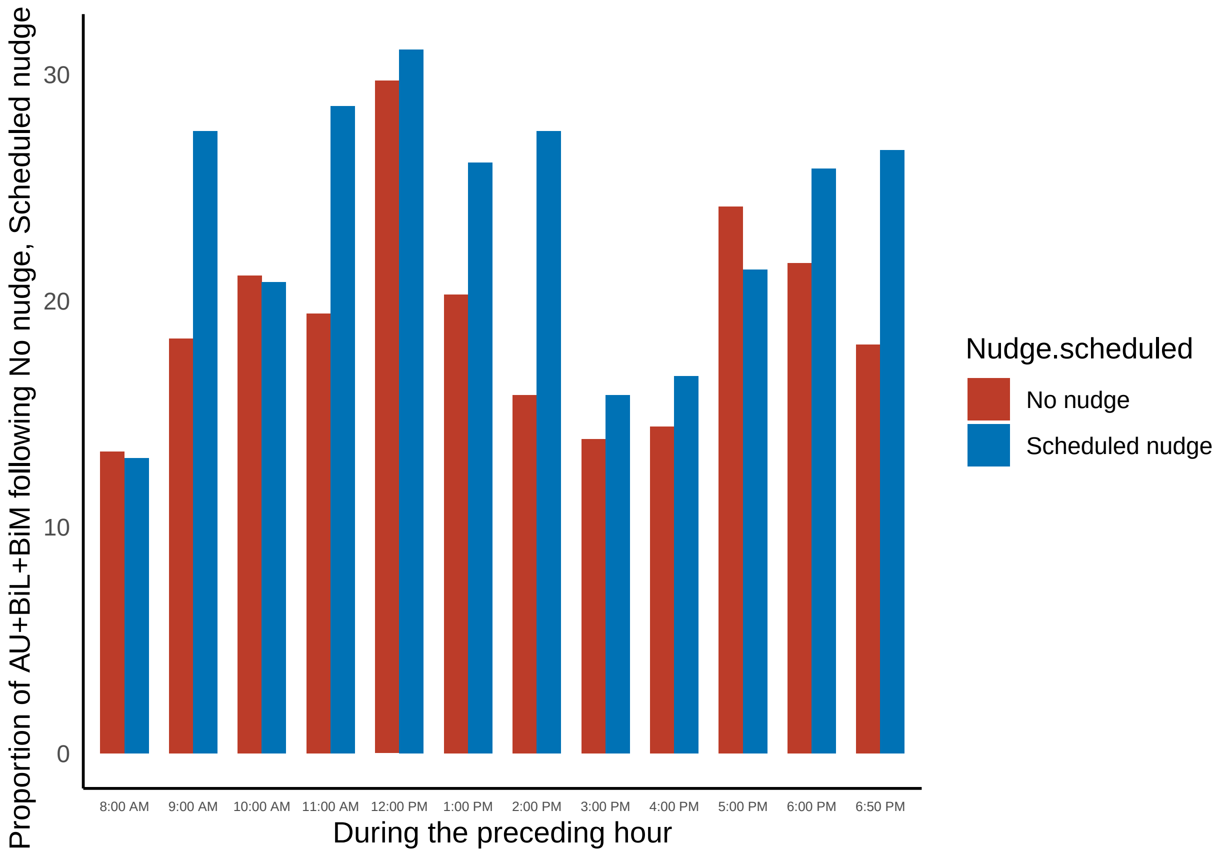

2.4 AU+BiL+BiM Proportions for Movement Observations over 10-second Intervals

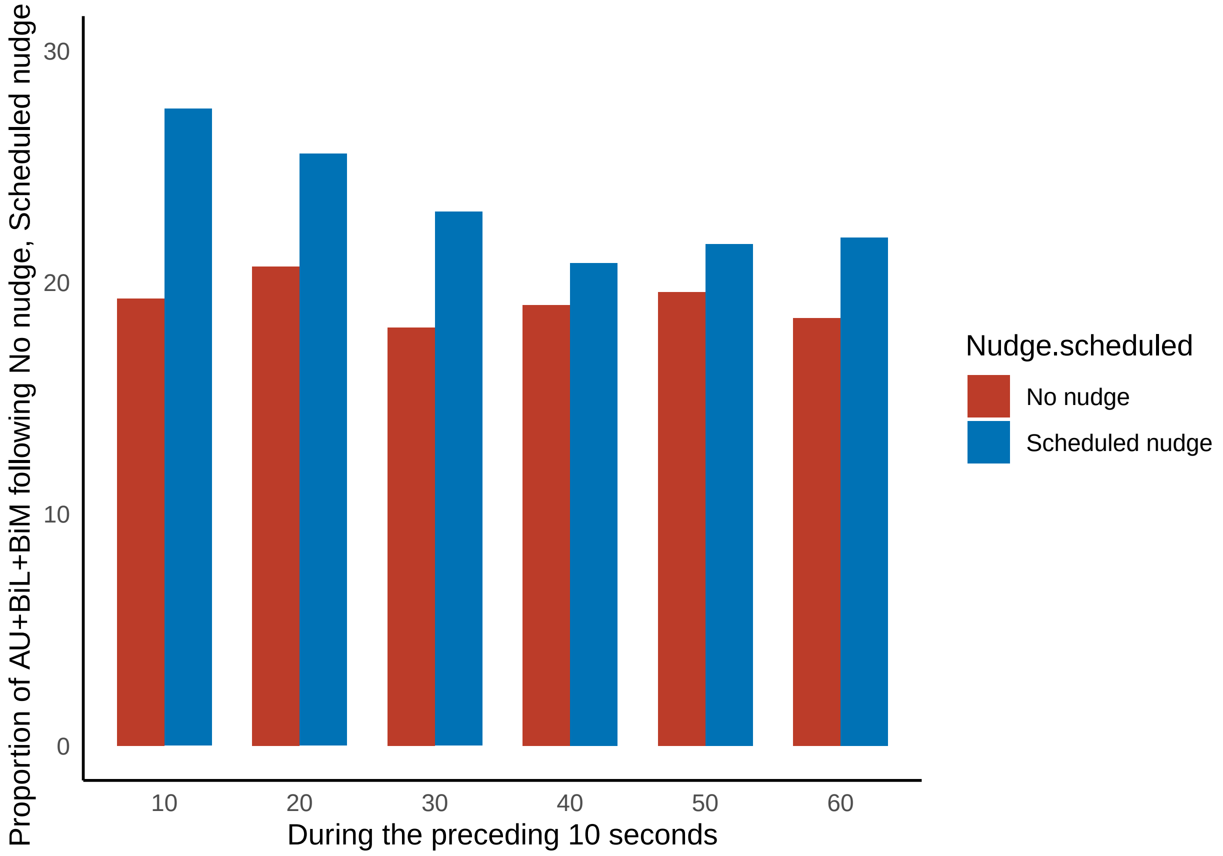

3 Instrumental Variable Analysis

3.1 Stage I: Confounder Estimation

3.1.1 Longitudnal Mixed Models

```
# Load saved models to avoid repeated computation costs
# during report generation
LOAD_SAVED <- 1

if(LOAD_SAVED == 1) {
  load(file = 'Model_IV_NR.RData')
  load(file = 'Model_IV_MS.RData')
  load(file = 'Model_IV_MO.RData')
} else {

  Model.IV.NR <- glmer(Nudge.received ~ (ns(Ten.minute.interval, knots = seq(7, 67, 6)) +
                                          More.of.the.same.scheduled +
                                          More.of.the.opposite.scheduled) *
                         Nudge.scheduled +
                         (1|ParticipantID/Hour),
                       family = binomial(link = "logit"),
                       Data.source,
                       control = glmerControl(optimizer = "bobyqa",
                                              optCtrl=list(maxfun = 1e6)))

  Model.IV.MS <- glmer(More.of.the.same ~ (ns(Ten.minute.interval, knots = seq(7, 67, 6)) +
                                            More.of.the.same.scheduled +
                                            More.of.the.opposite.scheduled) *
                         Nudge.scheduled +
                         (1|ParticipantID/Hour),
                       family = poisson(link = "log"),
                       Data.source,
                       control = glmerControl(optimizer = "bobyqa",
                                              optCtrl=list(maxfun = 1e6)))

  Model.IV.MO <- glmer(More.of.the.opposite ~ (ns(Ten.minute.interval, knots = seq(7, 67, 6)) +
                                                More.of.the.same.scheduled +
                                                More.of.the.opposite.scheduled) *
                         Nudge.scheduled +
                         (1|ParticipantID/Hour),
                       family = poisson(link = "log"),
                       Data.source,
                       control = glmerControl(optimizer = "bobyqa",
                                              optCtrl=list(maxfun = 1e6)))
}

# Obtain model residuals on the response scale

Data.source$NR.r <- resid(Model.IV.NR, type = "response")
Data.source$MS.r <- resid(Model.IV.MS, type = "response")
Data.source$MO.r <- resid(Model.IV.MO, type = "response")
```

3.2 Stage II: CASE Estimation

3.2.1 Data Imputations

```
# Random imputation at the level of the participant with the worst outcome

# Number of missing values following "No nudge"        = 542
Random.no.nudge         <- factor(rbinom(542, 1, 6.4814815/100),
                                  levels = c(0, 1), labels = c("UU+No", "AU+BiL+BiM"))

# Number of missing values following "Scheduled nudge"  = 580
Random.nudge.schedule   <- factor(rbinom(580, 1, 0.9259259/100),
                                  levels = c(0, 1), labels = c("UU+No", "AU+BiL+BiM"))

Data.source$Combined.UL.movement[is.na(Data.source$Combined.UL.movement) &
                                   Data.source$Nudge.scheduled ==
                                   "No nudge"]        <- Random.no.nudge
Data.source$Combined.UL.movement[is.na(Data.source$Combined.UL.movement) &
                                   Data.source$Nudge.scheduled ==
                                   "Scheduled nudge"] <- Random.nudge.schedule
```

3.2.2 Longitudnal Logistic Mixed Model

```
# Load saved models to avoid repeated computation costs
# during report generation
LOAD_SAVED <- 1

if(LOAD_SAVED == 1) {
  load(file = 'Model_IV.RData')
} else {
  Model.IV <- glmer(Combined.UL.movement ~ (ns(Ten.minute.interval, knots = seq(7, 67, 6)) +
                                             More.of.the.same +
                                             More.of.the.opposite +
                                             Ten.second.interval) * Nudge.received +
                      NR.r + MS.r + MO.r +
                      (1|ParticipantID/Hour),
                    data = Data.source,
                    family = binomial(link = "logit"),
                    control = glmerControl(optimizer = "bobyqa",
                                           optCtrl=list(maxfun = 1e6)))
}
```

4 Results

4.1 Immediate Effects of a Single Haptic Nudge on AUL Movement

```
Marginal.effects <- emmeans(Model.IV, ~ Nudge.received:
                              Ten.minute.interval:
                              More.of.the.same:
                              More.of.the.opposite:
                              Ten.second.interval,
                            at = list(Ten.minute.interval =
                                        c(seq(7, 12),
                                          seq(22, 27),
                                          seq(31, 36),
                                          seq(40, 45),
                                          seq(52, 57),
                                          seq(61, 66)),
                                      More.of.the.same = 0,
                                      More.of.the.opposite = 0,
                                      Ten.second.interval = 1),
                            type = "response")

Daytime.effects <- contrast(Marginal.effects,
                            list(Breakfast = # 8:00 AM to 9:00 AM
                                   c(rep(c(1/6, -1/6), 6), rep(0, 60)),
                                 Morning.activity = # 10:30 AM to 11:30 AM
                                   c(rep(0, 12), rep(c(1/6, -1/6), 6), rep(0, 48)),
                                 Lunch = # 12:00 PM to 1:00 PM
                                   c(rep(0, 24), rep(c(1/6, -1/6), 6), rep(0, 36)),
                                 Afternoon.activity = # 1:30 PM to 2:30 PM
                                   c(rep(0, 36), rep(c(1/6, -1/6), 6), rep(0, 24)),
                                 Rest = # 3:30 PM to 4:30 PM
                                   c(rep(0, 48), rep(c(1/6, -1/6), 6), rep(0, 12)),
                                 Dinner = # 5:00 PM to 6:00 PM
                                   c(rep(0, 60), rep(c(1/6, -1/6), 6)),
                                 Day = # 7:00 AM to 7:00 PM
                                   rep(c(1/36, -1/36), 36)))
```

Time of the day	Odds ratio	SE	95% CI lower	95% CI upper	Z-value	P-value
Breakfast	2.58	0.66	1.57	4.26	3.72	<0.001
Morning.activity	2.01	0.47	1.27	3.17	2.99	0.003
Lunch	2.17	0.53	1.35	3.49	3.20	0.001
Afternoon.activity	4.63	1.29	2.67	8.01	5.47	<0.001
Rest	2.51	0.67	1.49	4.22	3.45	<0.001
Dinner	1.36	0.32	0.86	2.16	1.30	0.194
Day	2.37	0.41	1.68	3.34	4.95	<0.001

Contrast	Ratio of odds ratio	SE	95% CI lower	95% CI upper	Z-value	P-value
Breakfast / Morning.activity	1.29	0.35	0.76	2.18	0.94	0.347

Contrast	Ratio of odds ratio	SE	95% CI lower	95% CI upper	Z-value	P-value
Breakfast / Lunch	1.19	0.32	0.71	2.00	0.66	0.51
Breakfast / Afternoon.activity	0.56	0.17	0.31	1.00	-1.97	0.049
Breakfast / Rest	1.03	0.29	0.60	1.79	0.11	0.913
Breakfast / Dinner	1.90	0.50	1.14	3.18	2.45	0.014
Breakfast / Day	1.09	0.19	0.78	1.52	0.50	0.614
Morning.activity / Lunch	0.92	0.26	0.53	1.61	-0.28	0.782
Morning.activity / Afternoon.activity	0.43	0.13	0.24	0.79	-2.76	0.006
Morning.activity / Rest	0.80	0.23	0.46	1.40	-0.78	0.435
Morning.activity / Dinner	1.48	0.39	0.88	2.47	1.49	0.137
Morning.activity / Day	0.85	0.15	0.60	1.20	-0.93	0.354
Lunch / Afternoon.activity	0.47	0.14	0.26	0.86	-2.46	0.014
Lunch / Rest	0.87	0.24	0.50	1.49	-0.52	0.604
Lunch / Dinner	1.60	0.41	0.97	2.64	1.83	0.067
Lunch / Day	0.92	0.16	0.65	1.29	-0.50	0.617
Afternoon.activity / Rest	1.85	0.57	1.01	3.37	2.00	0.046
Afternoon.activity / Dinner	3.41	1.01	1.90	6.10	4.12	<0.001
Afternoon.activity / Day	1.95	0.40	1.30	2.92	3.24	0.001
Rest / Dinner	1.84	0.55	1.03	3.29	2.07	0.038
Rest / Day	1.06	0.20	0.73	1.53	0.29	0.769
Dinner / Day	0.57	0.10	0.41	0.80	-3.23	0.001

4.1.1 Immediate Effects over the Day

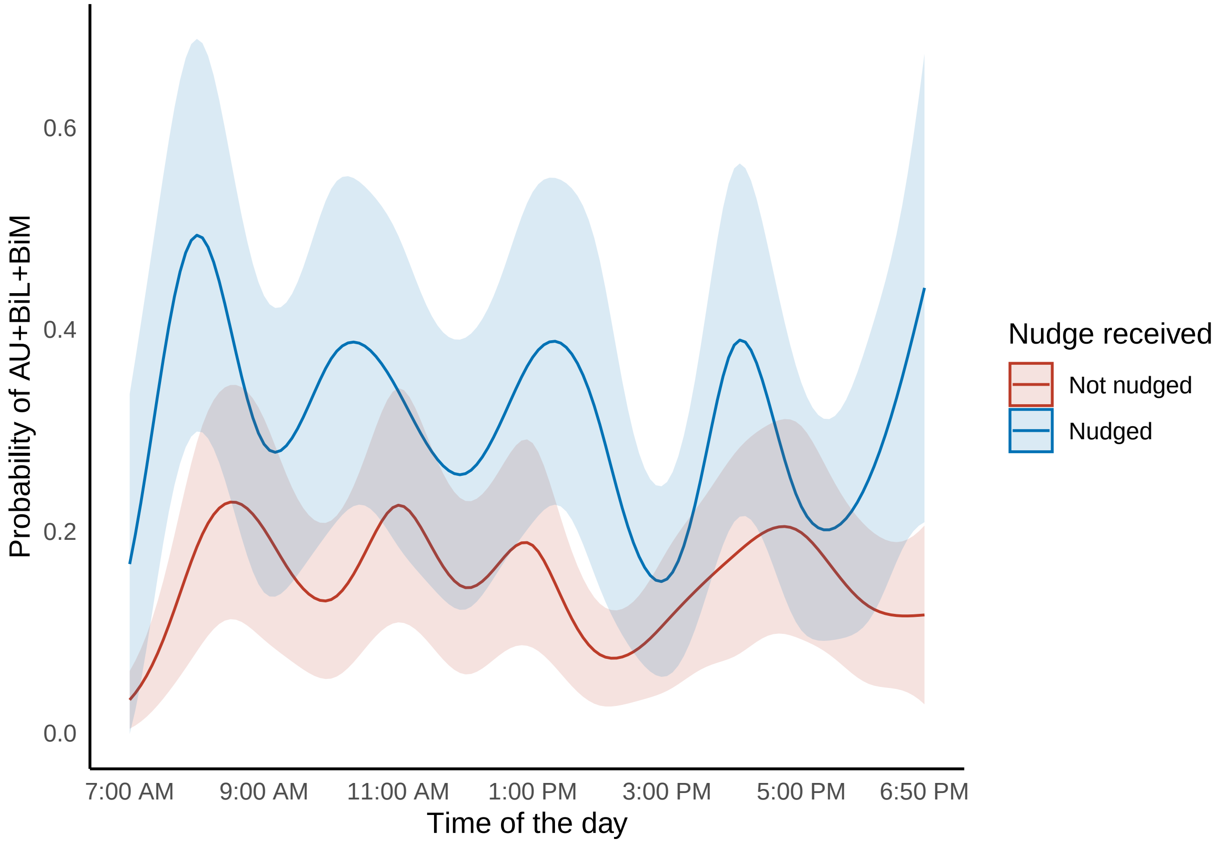

4.2 Late Effects of a Single Haptic Nudge on AUL Movement

```
Marginal.trends  <- emtrends(Model.IV, ~ Nudge.received,
                            var = "Ten.second.interval",
                            type = "response")

Marginal.effects <- emmeans(Model.IV, ~ Nudge.received:
                             Ten.minute.interval:
                             More.of.the.same:
                             More.of.the.opposite:
                             Ten.second.interval,
                           at = list(Ten.minute.interval =
                                     c(seq(7, 12),
                                       seq(22, 27),
                                       seq(31, 36),
                                       seq(40, 45),
                                       seq(52, 57),
                                       seq(61, 66)),
                                     More.of.the.same = 0,
                                     More.of.the.opposite = 0,
                                     Ten.second.interval = 6),
                           type = "response")

Daytime.effects <- contrast(Marginal.effects,
                           list(Breakfast = # 8:00 AM to 9:00 AM
                                  c(rep(c(1/6, -1/6), 6), rep(0, 60)),
                                Morning.activity = # 10:30 AM to 11:30 AM
                                  c(rep(0, 12), rep(c(1/6, -1/6), 6), rep(0, 48)),
                                Lunch = # 12:00 PM to 1:00 PM
                                  c(rep(0, 24), rep(c(1/6, -1/6), 6), rep(0, 36)),
                                Afternoon.activity = # 1:30 PM to 2:30 PM
                                  c(rep(0, 36), rep(c(1/6, -1/6), 6), rep(0, 24)),
                                Rest = # 3:30 PM to 4:30 PM
                                  c(rep(0, 48), rep(c(1/6, -1/6), 6), rep(0, 12)),
                                Dinner = # 5:00 PM to 6:00 PM
                                  c(rep(0, 60), rep(c(1/6, -1/6), 6)),
                                Day = # 7:00 AM to 7:00 PM
                                  rep(c(1/36, -1/36), 36)))
```

Nudge.received	Ten.second.interval.trend	SE	95% CI lower	95% CI upper	Z-value	P-value
Nudged	-0.11	0.03	-0.17	-0.06	-4.24	<0.001
Not nudged	0.00	0.02	-0.05	0.04	-0.14	0.887

Time of the day	Odds ratio	SE	95% CI lower	95% CI upper	Z-value	P-value
Breakfast	1.49	0.38	0.90	2.46	1.57	0.117
Morning.activity	1.16	0.27	0.73	1.83	0.63	0.526
Lunch	1.25	0.30	0.78	2.02	0.93	0.351
Afternoon.activity	2.67	0.75	1.54	4.62	3.51	<0.001

Time of the day	Odds ratio	SE	95% CI lower	95% CI upper	Z-value	P-value
Rest	1.45	0.39	0.86	2.44	1.39	0.166
Dinner	0.78	0.19	0.49	1.25	-1.02	0.306
Day	1.37	0.24	0.97	1.93	1.80	0.073

4.2.1 Late Effects over the Day

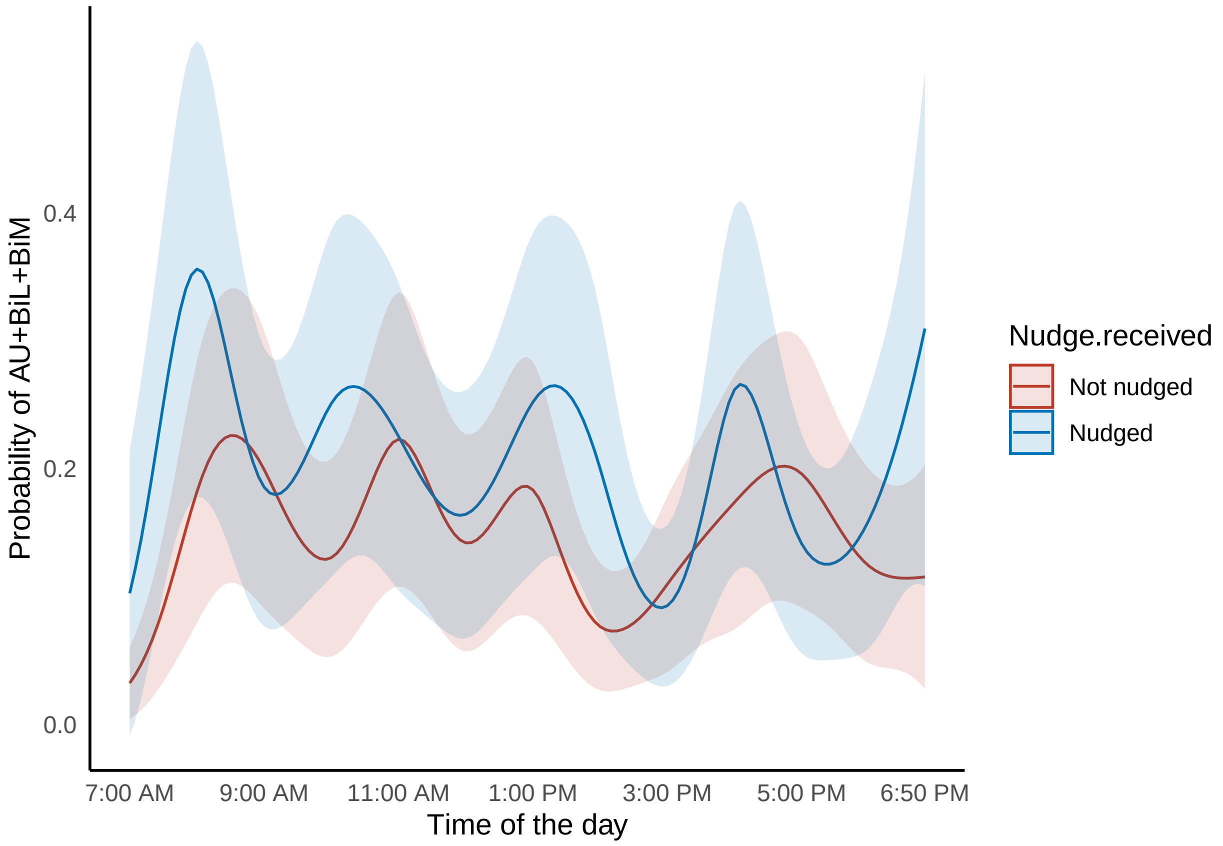

4.3 Effects of Repeated Nudges

```
Marginal.trends  <- emtrends(Model.IV, ~ Nudge.received,
                             var = "More.of.the.same",
                             type = "response")


Marginal.effects <- emmeans(Model.IV, ~ Nudge.received:
                              Ten.minute.interval:
                              More.of.the.same:
                              More.of.the.opposite |
                              Ten.second.interval,
                            at = list(Ten.minute.interval =
                                        c(seq(7, 12),
                                          seq(22, 27),
                                          seq(31, 36),
                                          seq(40, 45),
                                          seq(52, 57),
                                          seq(61, 66)),
                                      More.of.the.same = 2,
                                      More.of.the.opposite = 0,
                                      Ten.second.interval = c(1, 6)),
                            type = "response")

Daytime.effects <- contrast(Marginal.effects,
                            list(Breakfast = # 8:00 AM to 9:00 AM
                                   c(rep(c(1/6, -1/6), 6), rep(0, 60)),
                                 Morning.activity = # 10:30 AM to 11:30 AM
                                   c(rep(0, 12), rep(c(1/6, -1/6), 6), rep(0, 48)),
                                 Lunch = # 12:00 PM to 1:00 PM
                                   c(rep(0, 24), rep(c(1/6, -1/6), 6), rep(0, 36)),
                                 Afternoon.activity = # 1:30 PM to 2:30 PM
                                   c(rep(0, 36), rep(c(1/6, -1/6), 6), rep(0, 24)),
                                 Rest = # 3:30 PM to 4:30 PM
                                   c(rep(0, 48), rep(c(1/6, -1/6), 6), rep(0, 12)),
                                 Dinner = # 5:00 PM to 6:00 PM
                                   c(rep(0, 60), rep(c(1/6, -1/6), 6)),
                                 Day = # 7:00 AM to 7:00 PM
                                   rep(c(1/36, -1/36), 36)))
```

Nudge.received	More.of.the.same.trend	SE	95% CI lower	95% CI upper	Z-value	P-value
Nudged	-0.10	0.10	-0.3	0.11	-0.92	0.357
Not nudged	-0.23	0.09	-0.4	-0.06	-2.62	0.009

Time of the day	Im./Late	Odds ratio	SE	95% CI lower	95% CI upper	Z-value	P-value
Breakfast	Immediate	3.37	0.79	2.13	5.35	5.16	<0.001
Morning.activity	Immediate	2.62	0.65	1.61	4.26	3.89	<0.001
Lunch	Immediate	2.83	0.65	1.81	4.43	4.56	<0.001

Time of the day	Im./Late	Odds ratio	SE	95% CI lower	95% CI upper	Z-value	P-value
Afternoon.activity	Immediate	6.04	1.63	3.56	10.25	6.66	<0.001
Rest	Immediate	3.27	0.82	2.00	5.34	4.74	<0.001
Dinner	Immediate	1.77	0.42	1.11	2.83	2.40	0.016
Day	Immediate	3.09	0.51	2.25	4.26	6.90	<0.001
Breakfast	Late	1.95	0.46	1.23	3.09	2.82	0.005
Morning.activity	Late	1.51	0.38	0.93	2.46	1.67	0.095
Lunch	Late	1.64	0.37	1.04	2.56	2.15	0.032
Afternoon.activity	Late	3.49	0.94	2.05	5.92	4.62	<0.001
Rest	Late	1.89	0.47	1.16	3.09	2.54	0.011
Dinner	Late	1.02	0.25	0.64	1.64	0.10	0.922
Day	Late	1.79	0.29	1.29	2.47	3.52	<0.001

4.4 Effects of Deferred Nudges

```
Marginal.trends  <- emtrends(Model.IV, ~ Nudge.received,
                             var = "More.of.the.opposite",
                             type = "response")

Marginal.effects <- emmeans(Model.IV, ~ Nudge.received:
                              Ten.minute.interval:
                              More.of.the.same:
                              More.of.the.opposite |
                              Ten.second.interval,
                            at = list(Ten.minute.interval =
                                        c(seq(7, 12),
                                          seq(22, 27),
                                          seq(31, 36),
                                          seq(40, 45),
                                          seq(52, 57),
                                          seq(61, 66)),
                                      More.of.the.same = 0,
                                      More.of.the.opposite = 2,
                                      Ten.second.interval = c(1, 6)),
                            type = "response")

Daytime.effects <- contrast(Marginal.effects,
                            list(Breakfast = # 8:00 AM to 9:00 AM
                                   c(rep(c(1/6, -1/6), 6), rep(0, 60)),
                                 Morning.activity = # 10:30 AM to 11:30 AM
                                   c(rep(0, 12), rep(c(1/6, -1/6), 6), rep(0, 48)),
                                 Lunch = # 12:00 PM to 1:00 PM
                                   c(rep(0, 24), rep(c(1/6, -1/6), 6), rep(0, 36)),
                                 Afternoon.activity = # 1:30 PM to 2:30 PM
                                   c(rep(0, 36), rep(c(1/6, -1/6), 6), rep(0, 24)),
                                 Rest = # 3:30 PM to 4:30 PM
                                   c(rep(0, 48), rep(c(1/6, -1/6), 6), rep(0, 12)),
                                 Dinner = # 5:00 PM to 6:00 PM
                                   c(rep(0, 60), rep(c(1/6, -1/6), 6)),
                                 Day = # 7:00 AM to 7:00 PM
                                   rep(c(1/36, -1/36), 36)))
```

Nudge.received	More.of.the.opposite.trend	SE	95% CI lower	95% CI upper	Z-value	P-value
Nudged	-0.35	0.10	-0.53	-0.16	-3.62	<0.001
Not nudged	-0.05	0.09	-0.22	0.12	-0.55	0.582

Time of the day	Im./Late	Odds ratio	SE	95% CI lower	95% CI upper	Z-value	P-value
Breakfast	Immediate	1.42	0.35	0.87	2.32	1.41	0.157
Morning.activity	Immediate	1.11	0.29	0.66	1.84	0.38	0.701
Lunch	Immediate	1.19	0.29	0.74	1.93	0.73	0.465
Afternoon.activity	Immediate	2.55	0.72	1.46	4.44	3.29	0.001

Time of the day	Im./Late	Odds ratio	SE	95% CI lower	95% CI upper	Z-value	P-value
Rest	Immediate	1.38	0.36	0.82	2.31	1.22	0.223
Dinner	Immediate	0.75	0.19	0.46	1.22	-1.17	0.244
Day	Immediate	1.30	0.24	0.91	1.87	1.45	0.146
Breakfast	Late	0.82	0.21	0.50	1.34	-0.79	0.432
Morning.activity	Late	0.64	0.17	0.38	1.06	-1.72	0.086
Lunch	Late	0.69	0.17	0.43	1.12	-1.51	0.131
Afternoon.activity	Late	1.47	0.42	0.84	2.57	1.35	0.177
Rest	Late	0.80	0.21	0.47	1.34	-0.86	0.392
Dinner	Late	0.43	0.11	0.26	0.71	-3.34	<0.001
Day	Late	0.75	0.14	0.52	1.08	-1.53	0.126
